# Fingolimod Rescues Demyelination in a Mouse Model of Krabbe's Disease

**DOI:** 10.1523/JNEUROSCI.2346-19.2020

**Published:** 2020-04-08

**Authors:** Sibylle Béchet, Sinead A. O'Sullivan, Justin Yssel, Steven G. Fagan, Kumlesh K. Dev

**Affiliations:** Drug Development, School of Medicine, Trinity College Dublin D02 R590, Ireland

**Keywords:** fingolimod, FTY720, globoid cell leukodystrophy, Krabbe's disease, myelination, neurodevelopmental disease

## Abstract

Krabbe's disease is an infantile neurodegenerative disease, which is affected by mutations in the lysosomal enzyme galactocerebrosidase, leading to the accumulation of its metabolite psychosine. We have shown previously that the S1P receptor agonist fingolimod (FTY720) attenuates psychosine-induced glial cell death and demyelination both *in vitro* and *ex vivo* models. These data, together with a lack of therapies for Krabbe's disease, prompted the current preclinical study examining the effects of fingolimod in twitcher mice, a murine model of Krabbe's disease. Twitcher mice, both male and female, carrying a natural mutation in the galc gene were given fingolimod via drinking water (1 mg/kg/d). The direct impact of fingolimod administration was assessed via histochemical and biochemical analysis using markers of myelin, astrocytes, microglia, neurons, globoid cells, and immune cells. The effects of fingolimod on twitching behavior and life span were also demonstrated. Our results show that treatment of twitcher mice with fingolimod significantly rescued myelin levels compared with vehicle-treated animals and also regulated astrocyte and microglial reactivity. Furthermore, nonphosphorylated neurofilament levels were decreased, indicating neuroprotective and neurorestorative processes. These protective effects of fingolimod on twitcher mice brain pathology was reflected by an increased life span of fingolimod-treated twitcher mice. These *in vivo* findings corroborate initial *in vitro* studies and highlight the potential use of S1P receptors as drug targets for treatment of Krabbe's disease.

**SIGNIFICANCE STATEMENT** This study demonstrates that the administration of the therapy known as fingolimod in a mouse model of Krabbe's disease (namely, the twitcher mouse model) significantly rescues myelin levels. Further, the drug fingolimod also regulates the reactivity of glial cells, astrocytes and microglia, in this mouse model. These protective effects of fingolimod result in an increased life span of twitcher mice.

## Abstract (precis)

Krabbe's disease is an infantile neurodegenerative disease, which is affected by mutations in the lysosomal enzyme galactocerebrosidase, leading to the accumulation of its metabolite psychosine. We have shown previously that the S1P receptor agonist fingolimod (FTY720) attenuates psychosine-induced glial cell death and demyelination both *in vitro* and *ex vivo* models.

## Introduction

Krabbe's disease (KD; globoid cell leukodystrophy) is a devastating illness that is invariably fatal within the first 2 years of life (Graziano and Cardile, 2015). This disease has orphan status affecting ∼1:100,000 births, although the incidence varies in different populations (Barczykowski et al., 2012). Krabbe's disease is an inherited lipid storage disorder resulting from oligodendrocyte cell death and subsequent loss of myelin. The disease is caused by mutations in the *galc* gene encoding for galactosylceramidase (galc; Suzuki and Suzuki, 1970). Mutations in galc result in enzymatic dysfunction and a buildup of its two metabolites galactosylceramide and the toxic galactolipid galactosylsphingosine (psychosine; Suzuki, 1998). Aggregations of the latter are particularly apparent in the white matter (WM) of the brain and in sciatic nerves, where it has been shown to inhibit some critical cell processes resulting in oligodendrocyte and Schwann cell apoptosis (Giri et al., 2008; Misslin et al., 2017). Pathological features of Krabbe's disease therefore include profound demyelination and almost complete loss of oligodendrocytes in the white matter, accompanied by inflammatory mechanisms including reactive astrocytosis and infiltration of numerous multinucleated phagocytes termed “globoid cells” (Suzuki, 2003).

The clinical phenotype of Krabbe's disease is classified based on the age of disease onset, with the majority of cases affecting infants (Wenger et al., 2016). Infantile Krabbe's disease typically develops within the first 6 months postnatally with progressive rapid neurologic deterioration. Hallmark symptoms of the classic infantile forms include irritability, hypertonic spasticity, and psychomotor stagnation, followed by rapid developmental decline, seizures, and optic atrophy (Graziano and Cardile, 2015). Clinical manifestations thus suggest involvement of both the first and second motor neurons, indicative of a systemic disorder affecting the central as well as the peripheral nervous systems. To date, there is no therapeutic cure for Krabbe's disease. A number of therapeutic strategies have been described, targeting various levels of the pathomechanistic cascade to lower the psychosine load and reduce its neural toxicity (Bongarzone et al., 2016). The current standard of care for patients with Krabbe's disease is limited to hematopoietic stem cell transplantations, derived from bone marrow or umbilical cord blood (Escolar et al., 2005). While this treatment has been shown to slow disease progression, it fails to correct peripheral neuropathy in infants (Escolar et al., 2005; Duffner et al., 2009). With increasing evidence suggesting Krabbe's disease to be a multimodal illness, which includes ongoing inflammatory and neuronal pathologies, a combination of therapies targeting these processes may prove more promising.

Previously, we have shown that psychosine causes human and mouse astrocyte toxicity in culture, and demyelination in mouse organotypic slice cultures, effects that were attenuated by sphingosine 1-phosphate receptor (S1PR) agonists fingolimod and siponimod (O'Sullivan and Dev, 2015; O'Sullivan et al., 2016). S1PRs are G-protein coupled and expressed in many cell types, including immune system, cardiovascular system, and CNS (Dev et al., 2008). The drug fingolimod targets all five S1PR subtypes, apart from S1PR_2_ and is marketed as the first oral therapy for relapsing–remitting multiple sclerosis (Kappos et al., 2015). Fingolimod is described to work by internalizing S1PRs in T cells, thus limiting their egress from lymph nodes and dampening inflammation in multiple sclerosis (Dev et al., 2008). Furthermore, it has been extensively demonstrated that S1PRs regulate neuronal and glial cell function. Briefly, in glial cells, S1PRs play a role in oligodendrocyte differentiation, survival and myelination states, astrocyte cell migration, survival and cell signaling, microglia reactivity, and proinflammatory cytokine release (Osinde et al., 2007; Mullershausen et al., 2007; Dev et al., 2008; Miron et al., 2008, 2010; Mattes et al., 2010; Sheridan and Dev, 2012, 2014; Healy et al 2013; Pritchard and Dev 2013; O'Sullivan and Dev, 2013, 2015, 2017; O'Sullivan et al., 2016, 2017, 2018).

Given that fingolimod may have potential to alter both inflammatory and neuronal dysfunction in both the brain and periphery, and having previously shown that S1PR agonists attenuate psychosine-induced cell death of astrocytes and demyelination *in vitro*, the current study aimed to examine *in vivo* effects of fingolimod in twitcher mice, the murine model of Krabbe's disease.

## Materials and Methods

### 

#### 

##### Animals and experimental design.

A breeding colony of heterozygous twitcher mice was established in a pathogen-free environment in the Comparative Medicine Unit at Trinity College Dublin using mice obtained from the The Jackson Laboratory and maintained on a C57BL/6J genetic background (C57BL/6J-twi with C57BL/6J-twi). Homozygous and heterozygous animals were identified using genotyping. Heterozygous animals were used only for breeding purposes and were not used in the study. Fingolimod (SML0700, Sigma-Aldrich) was administered to both male and female wild-type and homozygous twitcher mice via drinking water (concentration, 6.25 μg/ml), which would result in a calculated final dose of 1 mg/kg/d.

##### Behavioral analysis.

All experiments were conducted in accordance with EU guidelines and authorized by the Health Products Regulatory Authority. All animals were subjected to a behavioral analysis beginning at 21 d of age [postnatal day 21 (P21)], on the day that treatment with fingolimod started. Behavioral observations were taken daily up to three times per day, and body weight was measured at each behavior testing time point. An observational scale was used to classify both the frequency and the severity of twitching during disease progression. For twitching frequency, a modified scoring system of a previously published protocol was used (Wicks et al., 2011). Very mild and fine twitching, similar to postweaning symptoms, were scored as score 1. Mild, intermittent fine twitching was given a score of 2, while constant fine twitching corresponded to a score of 3. Constant moderate twitching of body and head was given a score of 4, and was followed by killing if the mouse had a score of 4 during four independent observations. Constant and severe trembling and uncontrollable twitching was the highest score, followed immediately by humane killing. The locomotor deficits arising as a consequence of demyelination were quantified using a standardized manual scoring system that has been previously published and accepted for scoring mobility in animal models of multiple sclerosis (Beeton et al., 2007). A modified version of this scoring system, as per other previous publications using twitcher mice, was applied, with a minimum score of 1 indicating the absence of disease and a maximum score of 4. Due to overt phenotypic emergence in twitcher mice, the genotype of animals used in this study became evident during the study, which somewhat limited genotype-based blinding. Water bottles were filled with drug by the experimenter, and thus no blinding was included at the level of drug treatment. All data were randomized, and analysis was performed with data blinded to both genotype and drug treatment.

##### Processing of tissue for immunohistochemistry and immunoblotting.

Mice were killed by placing in a CO_2_ chamber. The spleen and kidneys were removed and placed in RIPA buffer, and animals were transcardially perfused with 20 ml of ice-cold PBS. Once perfusion was complete, animals were decapitated, and brains were removed and split into two hemispheres. One hemisphere was further separated into cerebellum and cortex. Together with spinal cord and sciatic nerves, cerebellum and cortex were stored in RIPA buffer supplemented with protease inhibitors at −80°C until processed for biochemical analysis. The remaining hemisphere was postfixed in 4% paraformaldehyde overnight (4°C) before being cryoprotected in 30% sucrose solution (4°C). Brains were then snap frozen in isopentane on dry ice and embedded in optimal cutting temperature compound (O.C.T.) Parasagittal cryosections of 12 μm thickness were cut and used for immunohistochemistry.

##### Histology and immunofluorescence.

Cerebellar cryosections were equilibrated to room temperature, and then rehydrated with PBS. Sections were permeabilized using 0.1% Triton X-100 (Tx; catalog #T9284, Sigma-Aldrich) in PBS to reduce nonspecific binding and were blocked with 10% bovine serum albumin (BSA; catalog #10735086001, Sigma-Aldrich) for 2 h at room temperature. Primary antibody incubations were conducted overnight at 4°C in PBS containing 2% BSA and 0.5% Tx. Primary antibodies used were anti-vimentin (1:1000 dilution; catalog #sc-373717, Santa Cruz Biotechnology), anti-glial fibrillary acidic protein (GFAP; 1:1000 dilution; catalog #ab7260, Abcam), anti-neurofilament heavy (NFH; 1;1000 dilution; catalog #MAB5539, Millipore), anti-SMI-32 (1:1000 dilution; Millipore), anti-myelin basic protein (MBP; 1:1000 dilution; catalog #ab40390, Abcam), anti-degraded MBP (dMBP; 1:1000 dilution; catalog AB5864, Millipore), anti-myelin oligodendrocyte protein (MOG; 1:1000 dilution; catalog #MAB5680, Millipore), anti-ionized calcium binding adapter molecule 1 (Iba1; 1:1000 dilution; catalog #ab5076, Abcam), and anti-calbindin (1:1000 dilution; catalog #ab108404, Abcam). Secondary antibody incubations were performed overnight at 4°C in PBS supplemented with 2% BSA and 0.05% Tx. Secondary antibodies used included Invitrogen goat anti-rabbit Alexa Fluor 488 (1:1000; A11008, Thermo Fisher Scientific), Invitrogen goat anti-chicken Alexa Fluor 633 (1:1000; A21103, Thermo Fisher Scientific), and Invitrogen anti-mouse DyLight 549 (1:1000; Thermo Fisher Scientific). A counterstain with Hoechst 33342 (1:10,000; catalog #62249, Thermo Fisher Scientific) labeling the nucleus was performed at the end of the immunofluorescence protocol. Slices were mounted and coverslipped on microscope slides and stored in the dark before being imaged. For periodic acid–Schiff (PAS; catalog #88016-88017, Thermo Fisher Scientific) and Luxol fast blue (LFB; catalog #10348250, Acros Organics) staining, the tissues were equilibrated to room temperature and rehydrated with PBS. LFB and PAS staining was performed according to standard histology procedures.

##### Light and fluorescence microscopy.

Data acquisition and quantification were similar to our previous studies (Sheridan and Dev, 2014). Confocal images captured for quantitative measurement of immunofluorescent staining were 12 bit.lif files of 1024 × 1024 or 2048 × 2048 pixel resolution and were randomly acquired throughout the cerebellum. They were captured using a Leica Sp8 scanning confocal microscope with 10× and 20× objectives. With five cerebellar slices per slide, ∼10–12 images were taken per slide to cover most of the total area of the cerebellum. Between six and eight slides were used per treatment group, making a minimum of 60 images analyzed per treatment group. Image acquisition settings were kept the same across different treatments per experiment. Image analysis was conducted using the software ImageJ (https://imagej.nih.gov/ij/). Regions of interest were manually selected on each image, and fluorescence intensity was averaged. Fluorescence intensity results are shown in arbitrary fluorescence units. For Iba1 quantification, we used *z*-stacks to compute 3D models of Iba1-positive microglia with Imaris software (Bitplane) rather than measuring fluorescence intensity. The surface of those 3D models was then used to identify area and volume taken up by microglia, with the hypothesis that the amoeboid and reactive state of microglia in twitcher mice will be reflected in higher volume measurements.

##### Immunoblotting.

Samples from cerebellum, spinal cord, and sciatic nerve were homogenized and sonicated in RIPA buffer containing protease inhibitors (cOmplete, catalog #11697498001, Roche), and centrifuged at 14,000 rpm, and supernatant was collected. For Western blotting, samples were denatured and electrophoresis was performed on 12% SDS-polyacrylamide gels. Electrophoresis was followed by a semi-dry transfer to a PVDF membrane (catalog #IPVH00010, Millipore), which was then blocked in 5% Marvel in 0.05% PBS/Tween 20 at room temperature. Incubation with primary antibody was performed overnight at 4°C. Primary antibodies used were rabbit anti-MBP (1:2000; catalog #ab40390, Abcam), mouse anti-MOG (1:1000; catalog# MAB5690, Millipore), rabbit anti-Olig2 (1:2000; catalog #ab109186, Abcam), mouse anti-Vimentin (1:1000; catalog #sc-373717, Santa Cruz Biotechnology), and chicken anti-GFAP (1:5000; catalog #ab7260, Abcam). The membranes were washed and incubated with secondary goat anti-rabbit (1:5000; catalog #NA934, VWR), or goat anti-mouse (1:5000; catalog #A16066, Thermo Fisher Scientific) HRP-conjugated antibodies for 2 h at room temperature. Membranes were developed using chemiluminescent HRP substrate (catalog #PIER34580, VWR), and images were acquired and analyzed on a C-Digit blot scanner with Image studio 4.0 (LI-COR).

##### Statistical analysis.

Experimental data were analyzed and graphically represented using GraphPad Prism 8.0 software package (GraphPad Software). The normality of the data was determined using the Shapiro–Wilk test. The difference in the life span between twitcher and wild-type mice was examined by Kaplan–Meier log-rank analysis. Discrepancies in weight gain, twitching severity, and mobility impairments were analyzed using the two-way ANOVA with Bonferroni *post hoc* test for multiple comparisons. Mean fluorescence intensity as measured with ImageJ software was used as an arbitrary unit of measure. Raw datasets were normalized and presented as percentages of the control group average. For that purpose, each arbitrary value in the experimental group was divided by the mean of the control group and multiplied by 100, to be expressed as a percentage value of the control average. Statistical evaluation of experimental data was performed with the two-way ANOVA followed by Bonferroni *post hoc* test, with *p* < 0.05 as the minimum level of significance. Graphical data are represented as the mean ± SEM.

## Results

### Fingolimod regulates levels of myelin in twitcher animals

**Figure 1.**
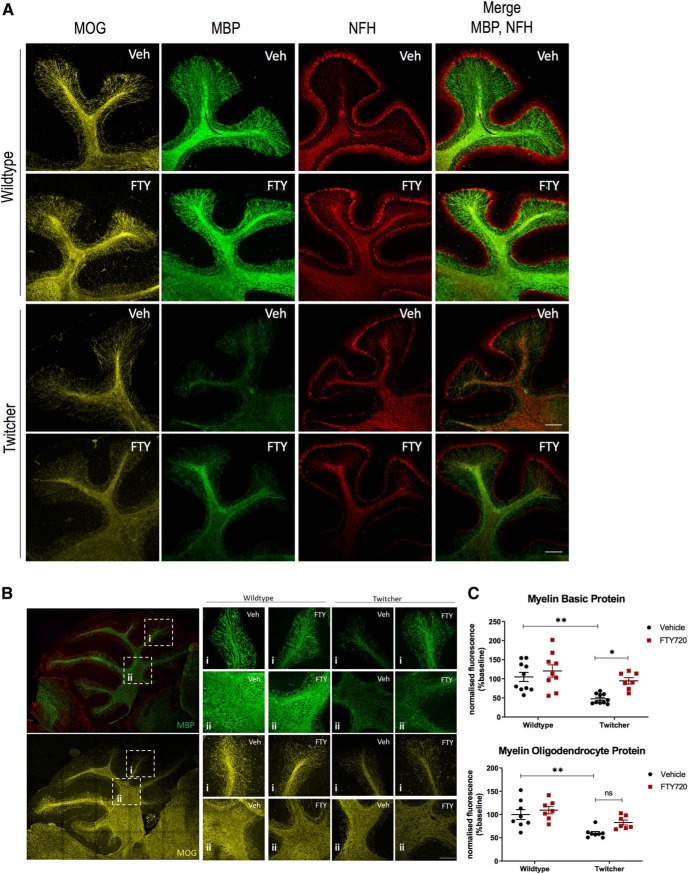
Levels of MBP in twitcher animals are rescued by fingolimod. ***A***, Cerebellar slices of vehicle- and FTY720-treated twitcher and wild-type mice were processed for immunohistochemistry. Representative images display MOG (yellow), MBP (green), and NFH (red) immunostaining under treatment conditions are indicated. Confocal images were captured at 10× magnification with focus on cerebellar lobes. Scale bar, 200 μm. ***B***, Confocal images were captured at 20× magnification with focus on inner white matter and lobe VII. Scale bar, 100 μm. ***C***, Graphs illustrating changes in MOG and MBP fluorescence post-treatment. Differences between treatment groups were analyzed using a two-way ANOVA followed by a Bonferroni correction for multiple comparisons (ns, not significant, *p < 0.05, **p < 0.01, *n* = 7–10 animals). Graphical data are represented as the mean ± SEM.

We have demonstrated that fingolimod promotes remyelination and inhibits demyelination in psychosine-treated cerebellar slice cultures, as well as in lysophosphatidylcholine-, H_2_O_2_-, and splenocyte-treated cerebellar slice cultures (Pritchard et al., 2014; Sheridan and Dev, 2014; O'Sullivan and Dev, 2015). Here, we examined myelin status in vehicle- and fingolimod-treated twitcher mice, using MBP and MOG, both of which constitute late markers of oligodendrocyte maturation and are involved in the final stages of myelin compaction. As expected, fingolimod did not alter the amount of myelin in wild-type littermates under nonpathological conditions (Fig. 1), an observation that coincides with previous findings (Miron et al., 2010). As anticipated, MBP and MOG immunostaining in vehicle-treated twitcher mice was significantly reduced compared with their wild-type littermates (MBP: 100 ± 11.23 vs 47.51 ± 3.72, **p = 0.0075, *n* = 10, df = 33; MOG: 100 ± 9.9 vs 59.97 ± 3.4, **p = 0.0036, *n* = 8, df = 26; Fig. 1). Importantly, we found the administration of fingolimod in twitcher mice increased significantly MBP expression compared with vehicle-treated animals (47.51 ± 3.72 vs 94.66 ± 7.78, *p = 0.04, *n* = 7–10, df = 33; Fig. 1), with no significant effect on MOG expression [59.97 ± 3.4 vs 82.75 ± 3.67, *p* = 0.248 (n.s.), *n* = 7–8, df = 26; Fig. 1].

**Figure 2.**
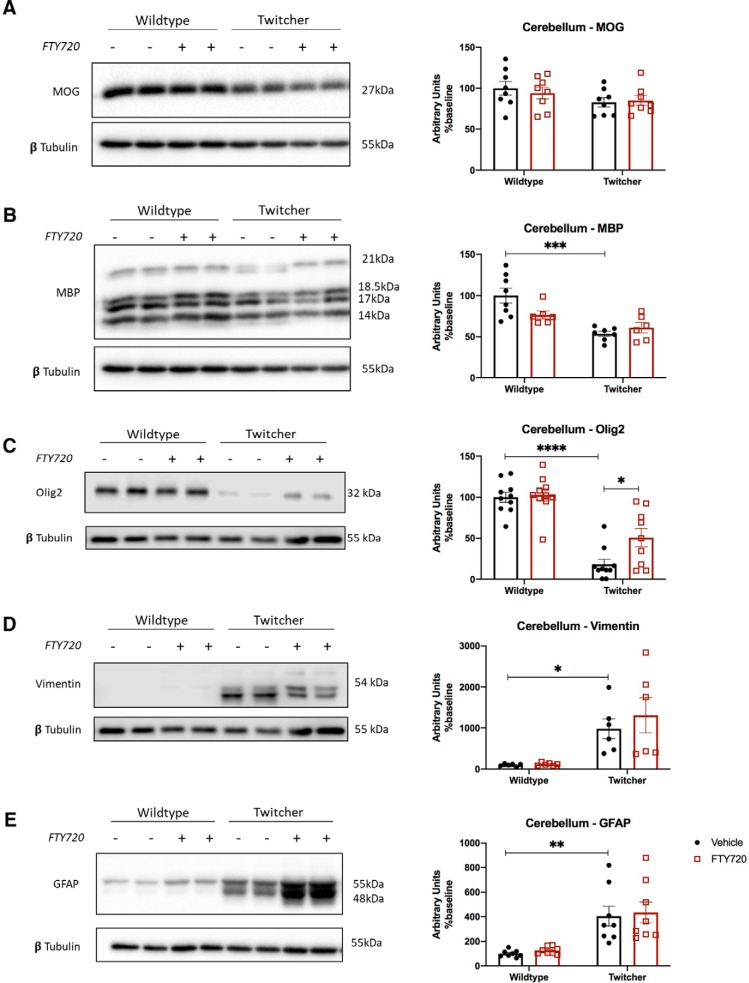
Changes in protein expression levels in the cerebellum of twitcher mice. Western blots on cerebellar tissue. ***A***, Total MOG remained at similar levels across all four treatment groups. ***B***, Data showing a significant decrease in total MBP levels in twitcher mice compared with wildtype littermates, with no significant effect of fingolimod. ***C***, Olig2 was reduced significantly in untreated twitcher mice, which was increased significantly with fingolimod treatment (*p < 0.05, **p < 0.01, ***p < 0.001, ****p < 0.0001, *n* = 10). ***D***, ***E***, Both vimentin (***D***) and GFAP (***E***) astrocyte markers were significantly increased in both treated and untreated twitcher mice. For vimentin, we noted the appearance of three independent bands, which, when individually analyzed, followed a pattern similar to that of the combined total levels shown in the figure. Graphical data are represented as the mean ± SEM.

**Figure 3.**
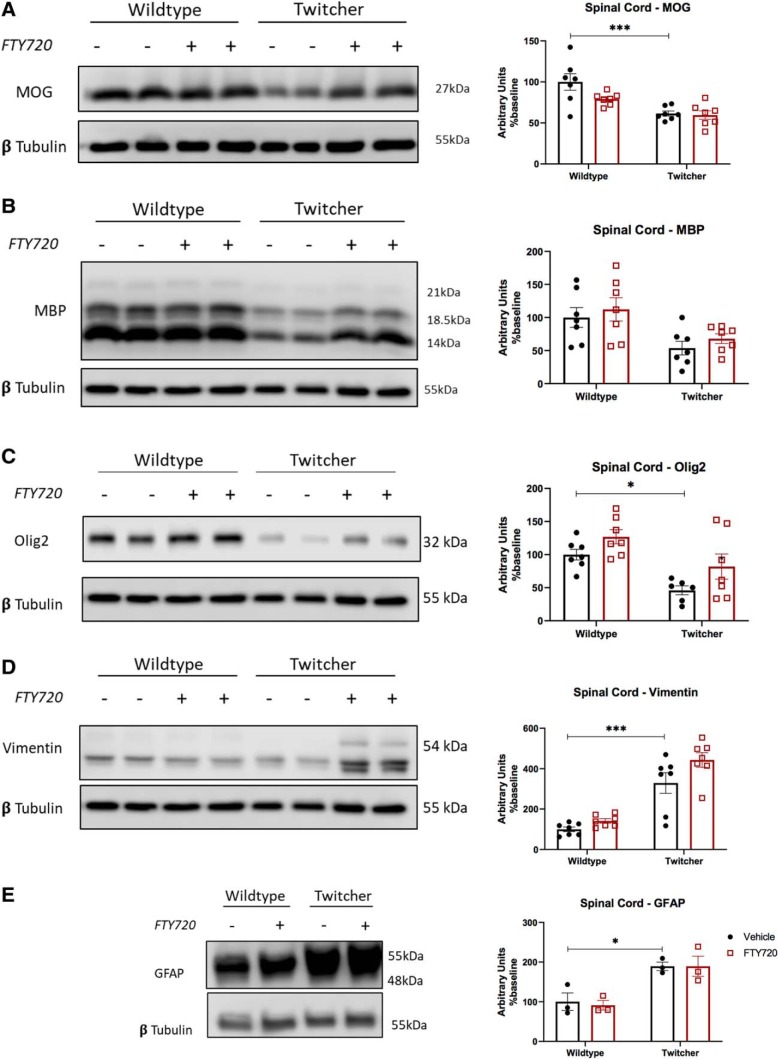
Changes in protein expression levels in the spinal cord of twitcher mice. Western blots on spinal cord tissue. ***A***, ***B***, Myelination as measured by MOG (***A***) and MBP (***B***) was significantly decreased in untreated twitcher mice, which was not altered with fingolimod treatment. ***C***, Olig2 was significantly reduced in untreated twitcher mice, with no significant effect in by fingolimod administration in twitcher mice (*p < 0.05, ***p < 0.001, *n* = 7). ***D***, ***E***, As observed in the cerebellum, the astrocyte markers vimentin (***D***) and GFAP (***E***) were significantly increased in both treated and untreated twitcher mice. Fingolimod did not significantly change protein levels of vimentin or GFAP in treated twitcher mice. Graphical data are represented as the mean ± SEM.

The effects of fingolimod on the total levels of MOG and MBP in wild-type and twitcher mice were also examined using homogenized cerebellum (Fig. 2*A*,*B*) and spinal cord (Fig. 3*A*,*B*) tissue by Western blotting. While, the levels of MBP in the cerebellum and MOG in the spinal cord were reduced in twitcher mice compared with vehicle controls, fingolimod did not alter the total expression levels of these markers (Figs. 2*A*,*B*, 3*A*,*B*). The expression levels of Olig2, which were significantly decreased in twitcher mice compared with wild-type littermates (Figs. 2*C*, 3*C*). In this case, fingolimod significantly enhanced the levels of Olig2 in twitcher mice compared with vehicle-treated controls in the cerebellum (Olig2: 18.209 ± 6.11 vs 50.684 ± 11.288, *p = 0.0386, *n* = 9–10, df = 35; Fig. 2*C*).

**Figure 4.**
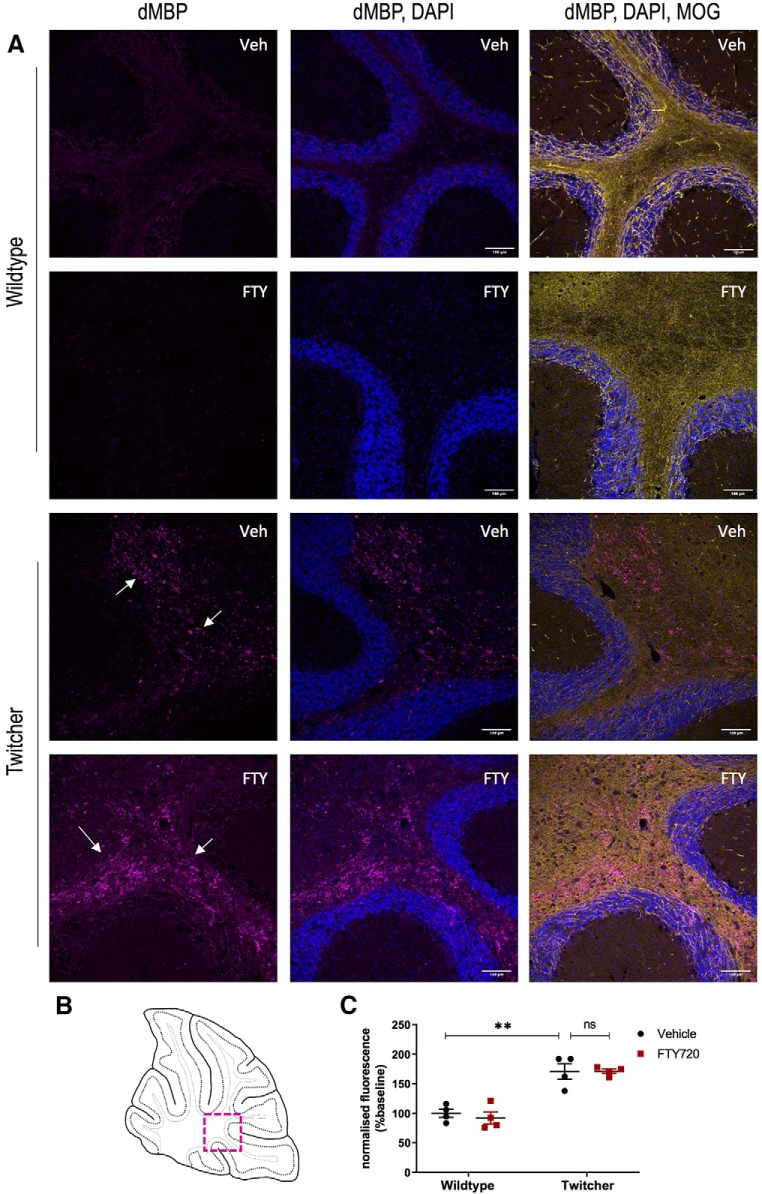
Impaired myelin debris clearance in the cerebellar white matter. ***A***, Representative images showing dMBP (purple), with the nuclear stain DAPI (blue) and MOG (yellow). Scale bar, 100 μm. ***B***, Images were taken at 20× magnification in the region of the cerebellar white matter, as depicted in the diagram (boxed region). ***C***, Graph illustrating changes in dMBP across treatment groups, with high levels of myelin debris in both treated and untreated twitcher mice compared with their healthy littermates (**p < 0.01; two-way ANOVA with Bonferroni's *post hoc* test; *n* = 4 animals, df = 10). ns, not significant.

To further investigate the effects of fingolimod, we also examined levels of myelin debris, by using an antibody that recognizes degraded MBP. As expected, the data showed a significant increase in levels of myelin debris in twitcher mice compared with wild-type controls (100 ± 7.3 vs 171 ± 13, **p = 0.0033, *n* = 4, df = 10; Fig. 4). We note here that the administration of fingolimod did not alter levels of myelin debris in wild-type or twitcher mice (171 ± 13 vs 171.3 ± 5.324, *p* > 0.9990, *n* = 4, df = 10; Fig. 4). Overall, the data indicate that fingolimod rescues demyelination in the brains of twitcher animals via a mechanism likely involving an increase in oligodendrocyte cells expressing MBP and Olig2, with little effect on total levels of myelin or on myelin debris.

### Fingolimod restores Bergmann glial cell expression and attenuates astrocyte activation

**Figure 5.**
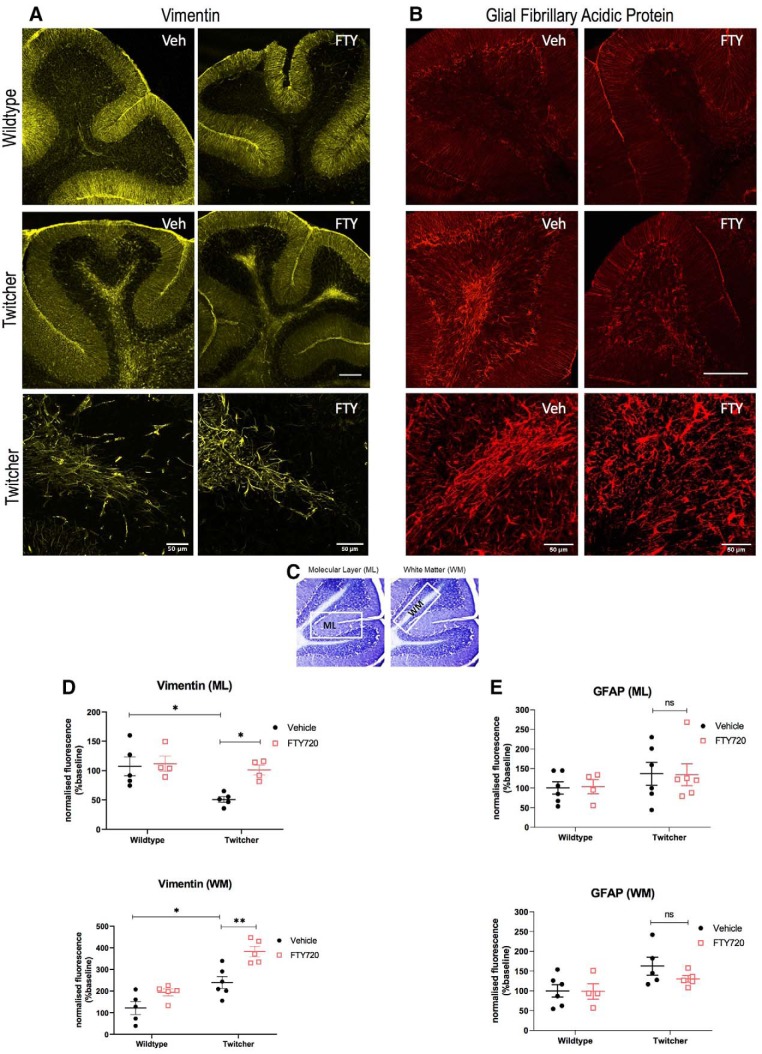
Fingolimod restores astrocyte reactivity in the cerebellum of twitcher mice. ***A***, ***B***, Representative images displaying vimentin (yellow; ***A***) and GFAP (red; ***B***) in the cerebellum of wild-type and twitcher mice under the treatment conditions indicated. Scale bar, 200 μm. Magnified images of twitcher mice are shown (lower panels). Scale bar, 50 μm. ***C***, Cresyl violet stain showing regions of interest analyzed, namely, ML and WM. ***D***, ***E***, Graphs illustrating vimentin (***D***) and GFAP (***E***) expression within the ML (top graphs) and WM (bottom graphs) of the cerebellum. Data are presented as the mean and median ± SEM. Differences between groups were analyzed using a two-way ANOVA followed by a Bonferroni correction for multiple comparisons (*p < 0.05, **p < 0.01, *n* = 5–6 animals). ns, not significant.

Psychosine induces death in human and mouse astrocytes *in vitro* that is attenuated by pretreatment with fingolimod (O'Sullivan and Dev, 2015). Here, we examined astrocytes using the type III intermediate filament astrocyte marker vimentin, which is involved in cytoskeleton formation in astrocytes (Fig. 5*A*), as well as the GFAP, which is enhanced following astrocyte activation (Fig. 5*B*). We differentiated between Bergmann glial cells in the molecular layer (ML) and fibrous astrocytes located in the WM, as depicted by cresyl violet staining (Fig. 5*C*). A significant decrease in vimentin fluorescence occurred between vehicle-treated wild-type and twitcher mice in the ML of the cerebellum (ML: 100 ± 14.86 vs 50.79 ± 4.42, **p = 0.0131, *n* = 5, df = 14; Fig. 5*D*). In agreement with our previous *in vitro* data, the administration of fingolimod promoted significantly the expression of vimentin in the molecular layer of twitcher mice, compared with their vehicle-treated littermates (ML: 50.79 ± 4.42 vs 90.32 ± 11.3, *p = 0.0394, *n* = 4–5, df = 14; Fig. 5*D*). Conversely, in the WM of twitcher mice, there was an increased vimentin fluorescence in vehicle-treated twitcher mice compared with control (WM: 100 ± 27.25 vs 239.83 ± 27.15, **p = 0.0067, *n* = 5–6, df = 16), which was further increased in fingolimod-treated twitcher animals (WM: 239.83 ± 27.15 vs 382.3 ± 24.18, **p = 0.0033, *n* = 5–6, df = 16; Fig. 5*D*). GFAP immunostaining showed no significant differences in the ML or WM layers of the cerebellum of untreated or fingolimod-treated twitcher mice compared with healthy controls [WM: 100 ± 15.87 vs 162.6 ± 20.75, *p* = 0.0699 (n.s.), *n* = 5, df = 16; ML: 100 ± 15.82 vs 136.644 ± 29.391, *p* > 0.9999 (n.s.), *n* = 4–6, df = 18; Fig. 3*E*]. Bergmann glia are generally aligned to Purkinje cell somata in ascending processes spanning the molecular layer. We noted that this organized astrocyte scaffold was disrupted in twitcher mice. Notably, however, higher-magnification images of vimentin and GFAP immunostaning did not show any overt qualitative differences in fibrous astrocyte morphologies. To examine whether fingolimod altered the total levels of vimentin and GFAP, Western blotting was performed using homogenized cerebellum (Fig. 2*D*,*E*) and spinal cord (Fig. 3*D*,*E*). While the levels of vimentin and GFAP increased in twitcher mice compared with vehicle controls, fingolimod treatment did not alter the total levels of these markers (Figs. 2*D*,*E*, 3*D*,*E*). Overall, these data suggest that astrocytes have an altered function in the cerebellum of twitcher mice, and this is modulated by fingolimod.

### Fingolimod decreases microglia levels in the cerebellar white matter of twitcher mice

**Figure 6.**
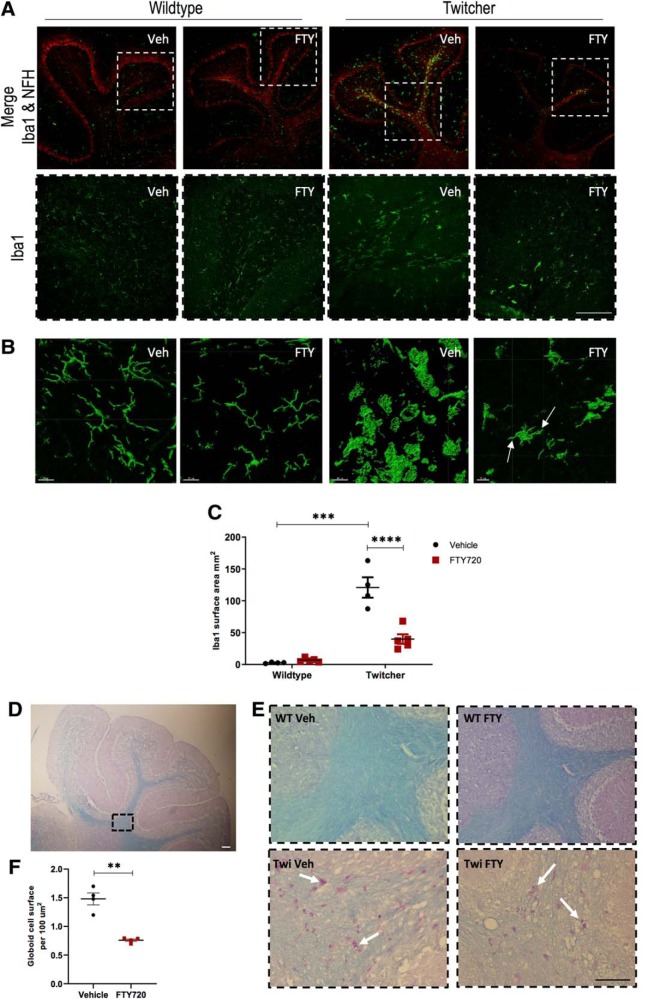
Fingolimod attenuates Iba1 expression in the cerebellum of twitcher mice. ***A***, Representative images of Iba1 (green) and NFH (red) immunostaining. Iba1 was significantly increased in untreated twitcher mice, with accumulations centered around the white matter. ***B***, Magnified images created using IMARIS software are shown. Scale bar, 20 μm. ***C***, Graph illustrates Iba1-positive areas expressed in square millimeters. Data are presented as the mean ± SEM. Differences between treatment groups were analyzed using a two-way ANOVA followed by a Bonferroni correction for multiple comparisons (*n* = 4–5 animals). ***D***, Low-magnification representative image of PAS-stained globoid cells (purple) accumulating along white matter tracts (blue). ***E***, High-magnification representative images of PAS stain, from region indicated in dotted box. Scale bar, 200 μm. ***F***, Graph illustrates globoid cell surface per 10 μm^2^ in twitcher mice treated with vehicle or fingolimod. Differences analyzed with an unpaired *t* test, *p* < 0.01 (*n* = 4 animals). Graphical data are represented as the mean ± SEM. **p < 0.01, ***p < 0.001, ****p < 0.0001.

The CNSs of patients with KD, as well as those of animal models of KD, exhibit robust neuroinflammation with microglial activation and the presence of phagocytic multinucleated globoid cells (Potter and Petryniak, 2016). Fingolimod possesses potent anti-inflammatory effects in various animal models of CNS injury and neurodegeneration (Aytan et al., 2016; Martinez and Peplow, 2018). Furthermore, fingolimod shifts LPS-activated microglia toward a neuroprotective microglial phenotype (Cipriani et al., 2015). We therefore investigated the effect of fingolimod treatment as a neuroinflammatory modulator in twitcher mice. Surprisingly, in our hands, we have not observed psychosine to alter the expression of Iba1 in organotypic cerebellar slice cultures (O'Sullivan and Dev, 2015), although we note that Iba1 is not a specific marker of altered microglia reactivity. In contrast to this, and in line with our expectations, here we found a significant increase in Iba1 immunostaining in vehicle-treated twitcher mice, compared with their wild-type littermates (2.675 ± 0.5 vs 120.9 ± 16.02, *p* < 0.0001, *n* = 5, df = 13; Fig. 6*A–C*). More importantly, fingolimod significantly decreased Iba1 expression in twitcher mice compared with vehicle-treated controls (120.9 ± 16 vs 32.93 ± 3.5, *p* < 0.0001, *n* = 5, df = 13; Fig. 6*A–C*). Higher-magnification images of Iba1 immunostaining showed amoeboid morphology in twitcher mice compared with wild-type littermates, which appeared less so in twitcher mice administered fingolimod, although these microglia also still appeared very much of an amoeboid structure (Fig. 6*B*).

Multinucleated phagocytes are a histopathological hallmark of KD and have been shown to accumulate large amounts filamentous inclusions that stain positively for periodic acid–Schiff stain. The distribution of these globoid cells in the cerebellar white matter, of treated and untreated twitcher mice, was examined also by staining with Luxol fast blue and periodic acid–Schiff stains. Our data showed increased levels of periodic acid–Schiff staining in vehicle-treated twitcher mice (1.48 ± 0.25 × 100 μm^2^), which was reduced significantly in fingolimod-treated animals (0.75 ± 0.05 × 100 μm^2^; Fig. 6*D–F*). These observations demonstrate increased Iba1 expression and increased globoid cell expression in twitcher mice, which is attenuated significantly by fingolimod.

### Effects of fingolimod on phosphorylation state of neurofilaments and Purkinje cells

**Figure 7.**
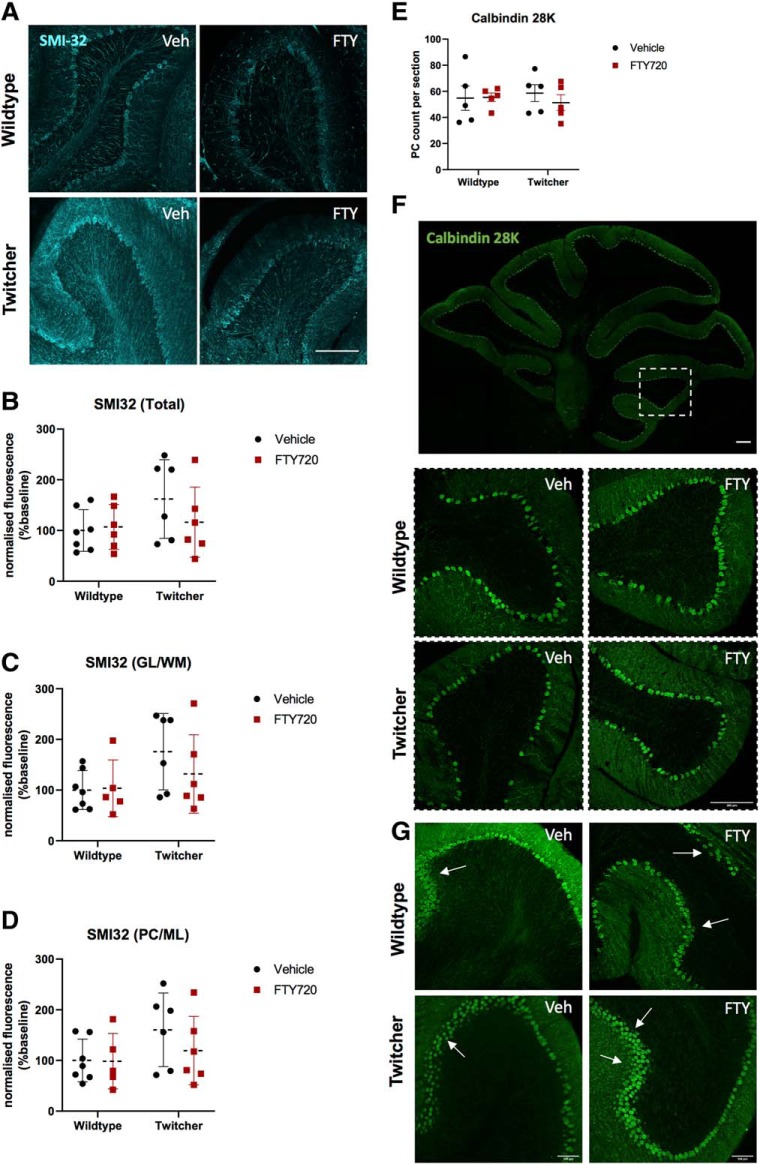
Fingolimod did not alter axonal damage or Purkinje cell layer architecture in twitcher mice. ***A***, Representative images of SMI32 immunostaining. Confocal *z*-stacks were captured at 20× magnification. Scale bars, 200 μm. ***B–D***, Graphical data showing SMI32 fluorescence intensity measurements, which were taken across the different layers of the cerebellar cortex (*n* = 6–7 animals). ***E***, Graphical data showing Purkinje cell count did not change across treatment groups (*n* = 5 animals). Data are represented as the mean ± SEM. ***F***, Low-magnification representative image of Calbindin immunostaining of Purkinje cells. Dotted box, High-magnification representative images of Calbindin immunostaining. Confocal *z*-stacks were captured at 20× magnification. Scale bars, 200 μm. ***G***, Representative images showing presence of heterotopic layers of Purkinje cells in vehicle- and FTY720-treated twitcher mice and to a certain degree in healthy wild-type animals. Scale bars, 100 μm.

Neurofilaments are neuron-specific cytoskeletal proteins that are essential for the development and maintenance of neurons and their processes. They form structurally and genetically related protein subunits, which are thought to be associated with the level of myelination and to be related to the fast conduction of axons (Yuan et al., 2012). Previous studies have shown that nonphosphorylated NFH epitopes are expressed in neuronal cell bodies and Purkinje cells under physiological conditions (Demilly et al., 2011). Increased expression of nonphosphorylated NFH in axonal tracts, however, has been associated with impaired axonal conduction, demyelination, and neuronal damage (Misslin et al., 2017). To examine the NFH phosphorylation state in twitcher mice, cerebellar slices were stained for NFH and SMI-32, a marker known to label nonphosphorylated NFH. A qualitative observation showed increased expression of SMI32 in twitcher animals, as expected, which was decreased in fingolimod-treated twitcher mice, as reflected by a change in the mean quantification of SMI32 fluorescence intensity. We note, however, a level of variance in the expression of SMI32 across animals, and more so in the twitcher animals compared with wild-type controls, which resulted in a nonsignificant statistical analysis (Fig. 7*A–D*). Cerebellar Purkinje cells are pivotal for motor coordination, and their dysfunction generally leads to ataxia (Hoxha et al., 2018). The ataxic tremors and hindlimb paralysis observed in human and murine KD suggests the pathological involvement of Purkinje cells within cerebellar degeneration. Given that little is known regarding the pathology of the Purkinje cell layer in KD, we investigated the number of Purkinje cells in this layer by immunostaining with Calbindin. No overall difference in the number of calbindin-positive Purkinje cells was observed (Fig. 7*E*) in twitcher mice compared with wild-type littermates and compared with twitcher mice treated with fingolimod (Fig. 7*E*,*F*). Structural changes were observed, including the presence of heterotopic layers of Purkinje cells of fingolimod- and vehicle-treated twitcher mice, with their soma displaced in the molecular layer (Fig. 7*G*). These data suggest that neuronal damage in twitcher mice is not necessarily driven by Purkinje cell loss, but rather by their abnormal architectural integrity.

### Fingolimod enhances life span of twitcher mice

**Figure 8.**
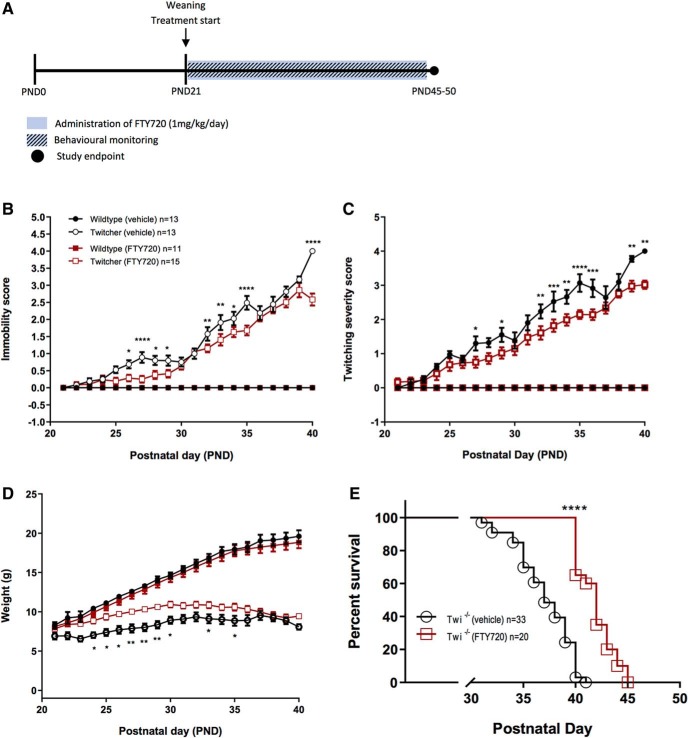
Fingolimod enhances the life span of twitcher mice. ***A***, Schematic representation of the study design. Fingolimod treatment commenced at P21 at a concentration of 1 mg/kg/d with daily behavior monitoring. ***B–D***, Mobility (***B***), twitching (***C***), and body weight (***D***) were assessed statistically using a two-way ANOVA followed by a Bonferroni adjustment for multiple comparisons (*p < 0.05, **p < 0.01, ***p < 0.001, ****p < 0.0001, *n* = 11–15 animals). ***E***, The Kaplan–Meier survival curve illustrates the life span of fingolimod-treated versus untreated twitcher mice. A log-rank Mantel–Cox test reveals that both treatment groups were significantly different in their survival distribution (****p < 0.0001, twitcher mice treated with fingolimod vs untreated, *n* = 20–33 animals), with an increased average life span of FTY720-treated twitcher mice when compared with their vehicle-treated twitcher littermates. Graphical data are represented as the mean ± SEM.

The administration of fingolimod from P21 onward (Fig. 8*A*) in twitcher mice significantly improved their mobility (Fig. 8*B*) and twitching severity (Fig. 8*C*), which we noted had relapsing–remitting trends. Fingolimod also positively affected body weight, with significant improvement between P25 and P30 (Fig. 8*D*), although this weight gain plateaued at P30 and finally converged toward vehicle-treated animals. Importantly, Kaplan–Meier survival curves demonstrated fingolimod to modestly but significantly increase the life spans of twitcher mice versus vehicle-treated littermates (Fig. 8*E*). We noted that this effect showed an ∼10% increase in average life span of fingolimod-treated (42 d) versus vehicle-treated (37 d) twitcher mice (Fig. 8*E*). While we had hoped for a more promising effect on life span, this result reflects the well known multiorgan involvement of leukodystrophies, where neuroprotective effects of drugs such as fingolimod may become an important part of a combination therapy that targets both central and peripheral systems in these illnesses.

## Discussion

### Summary of findings

The rapid and complete loss of myelin and myelin-forming oligodendrocytes is a predominant pathological feature of KD, and is responsible for slowed nerve conduction observed in twitcher animals, a natural occurring animal model of KD (Davenport et al., 2011). With studies reporting remyelinating effects of fingolimod in relapsing–remitting MS (Münzel et al., 2013), and, based on our previous *in vitro* work demonstrating fingolimod-mediated protective effects against astrocyte cell toxicity and demyelination, we anticipated that this drug would display efficacy in other demyelinating diseases, such as KD. Here, we investigated the efficacy of fingolimod treatment on phenotypic and underlying biochemical abnormalities in twitcher mice, the murine model of KD. The present results enabled a direct demonstration that fingolimod can rescue deficits seen at the pathological level, namely levels of demyelination, as well as promote life span and attenuate phenotypic dysfunction in these animals.

### The regulation of oligodendrocytes by fingolimod

With fingolimod being able to cross the blood–brain barrier and act on S1PRs within the CNS, this drug has been postulated to enhance and stabilize myelination by direct action on oligodendrocyte lineage cells through S1PR_1,3,5_ subtypes (Miron et al., 2010). Specifically, mature oligodendrocytes express high levels of S1PR_5_, while oligodendrocyte precursor cells (OPCs) show preferred expression of S1PR_1_ (Choi and Chun, 2013). Moreover, remyelination requires OPCs to proliferate, migrate to sites of demyelination, and finally to differentiate into mature myelin-forming oligodendrocytes, in which S1PRs play a role (Miron et al., 2011). The observed enhanced Olig2 expression in cerebellar samples, in conjunction with increased expression of the myelination marker MBP induced by fingolimod may suggest a remyelination event that involves an increase in OPC numbers, although we note the caveat that Olig2 is not expressed only in OPCs. Treatment with pFTY720 also regulates the cell survival and differentiation in oligodendrocyte lineage cells in cultured rat oligodendrocytes and OPCs (Jaillard et al., 2005; Jung et al., 2007). Additionally, S1P1 activation is involved in OPC mitogenesis (Jung et al., 2007). In a cuprizone model of demyelination, fingolimod increases the number of OPCs, without, however, promoting remyelination (Kim et al., 2011). Importantly, pFTY720 modulates mature oligodendrocyte membrane dynamics and survival responses in human oligodendrocyte cultures (Miron et al., 2008). Together, these findings support the possibility of an immediate protective effect of S1PR modulation on oligodendrocytes through various signaling pathways.

### Fingolimod regulates microglia in twitcher mice

The psychosine hypothesis was introduced in 1972 and attributes the underlying cellular and biochemical pathology of KD to supraphysiological aggregations of psychosine in the periphery and CNS (Suzuki, 1998). Emerging evidence complements this long-standing hypothesis by ascribing early demise in KD to neuroinflammation rather than myelin loss (Nicaise et al., 2016). In addition to fingolimod positively regulating the levels of myelin in twitcher mice, our current study supports the involvement of immunomodulation via glial cells. The activation of astrocytes and microglia have been reported to play a compounding role in the disease progression of KD (Mohri et al., 2006; Ijichi et al., 2013). Microglia mediate inflammation through increased levels of prostaglandin (PG) D2, while astrocytes increase PGD2 receptor expression (Mohri et al., 2006). This study reported suppressed astrogliosis and reduced demyelination when blocking PGD2 synthase in twitcher mice (Mohri et al., 2006). The remyelination in fingolimod-treated twitcher animals observed in our current study coincides with the reduction we observed in the immunostaining of Iba1, albeit as surrogate marker of microglia reactivity. These findings correlate with the hypothesis that the regulation of S1PRs mediates enhanced levels of myelination via a mechanism that involves immunomodulatory pathways and microglia (Ijichi et al., 2013).

### The S1P axis regulates astrocytes and astrogliosis in twitcher mice

A growing body of evidence in KD research paints a complex picture of combined axonal, neuronal, and myelination abnormalities in the CNS and periphery, accompanied by exacerbating astrogliosis and neuroinflammatory events (Nicaise et al., 2016; Won et al., 2016). Dysfunctional astrocytes may be a contributing factor to disease progression of various neurological disorders (Molofsky et al., 2012). Astrocytes are implicated in the pathogenesis of KD, where the astrocytic expression of matrix metalloproteinase-3 is elevated at symptomatic onset in twitcher mice, continues to rise with disease progression, and potentially induces the formation of multinucleated globoid cells and activated phagocytes (Ijichi et al., 2013; Claycomb et al., 2014). Such findings coincide with results from our current study, where immunofluorescence in twitcher mice shows altered the expression of vimentin in areas of demyelination, accompanied by PAS-positive globoid cells. Our data showed that Bergmann glial cells in the molecular layer of the cerebellum stained with vimentin were reduced in twitcher mice and that this was attenuated by treatment with fingolimod. In contrast, the fibrous astrocytes populating the white matter showed increased expression of vimentin in twitcher mice, which was further enhanced by treatment with fingolimod. Both GFAP and vimentin are highly expressed in white matter astrocytes, but poorly label gray matter astrocytes (Molofsky et al., 2012). The differential expression of these intermediate filaments suggests that fibrous astrocytes serve a specialized function with regard to structure among myelinated fibers (Lundgaard et al., 2014). We note that in our study, we did not observe statistical differences with GFAP staining between wild-type and twitcher mice treated with or without fingolimod. This could be explained by the fact that GFAP is expressed late in the development of fibrous astrocytes, and is therefore not an adequate marker to investigate changes during early developmental stages (Lundgaard et al., 2014). Supporting the role of S1PRs in astrocytes, pFTY720 attenuates psychosine-induced apoptosis in human astrocyte cultures and proinflammatory cytokines in murine astrocyte cultures (O'Sullivan and Dev, 2015). The selective deletion of S1PR1 from astrocytes also reduces experimental autoimmune encephalomyelitis (EAE)-related phenotypic severity and histological abnormalities, including demyelination and astrogliosis (Choi et al., 2011). These previous studies, together with our own, could be interpreted as a pFTY720-induced phenotypic change in astrocytes.

### Regulation of neuronal dysfunction through S1P modulation

In addition to affecting oligodendrocytes, psychosine also impairs axonal transport (Cantuti-Castelvetri et al., 2012). Neurons are not known to synthesize significant quantities of psychosine, which is likely transferred from glial cells, resulting in neuronal damage and impaired axonal conduction. Psychosine accumulation increases PP1 and PP2A phosphatases, inducing neurofilament dephosphorylation and inhibiting axonal transport (Cantuti-Castelvetri et al., 2012). Axonopathy precedes demyelination and neuronal apoptosis in twitcher mice, with neuronal damage occurring at later stages of disease progression (Castelvetri et al., 2011; Smith et al., 2011). Importantly, pFTY720 has protective effects on neuronal function and axonal integrity by reducing axonal damage following demyelination, for example, in a cuprizone model or by regulating glutamatergic transmission in EAE mice (Slowik et al., 2015). These findings coincide with our observations using SMI32 as a marker for axonal damage, where we observed a qualitative decrease in the expression of SMI32 in twitcher animals administered with fingolimod, although we note that this was not reflected by a quantitative difference. Nevertheless, the current data are in line with previous observations that suggest S1PR modulation involves a concurrent preservation of axonal damage as well as a restoration of myelin levels.

### S1P modulation and its impact on the cerebellar Purkinje cell layer architecture

A striking pathological hallmark of KD encompasses cerebellar ataxia, found in 57% of infantile-onset cases (Debs et al., 2013). As disease progresses, twitcher mice display tremors, ataxia, and severe hindlimb weakness. Such motor deterioration suggests the involvement of cerebellar degeneration in addition to pyramidal tract dysfunctions. Our results displayed structural changes in the cerebellum of twitcher mice that appear to be characterized by a pruning of dendritic arborization and heterotopic layers of Purkinje cells. These findings are in agreement with those of other studies showing exacerbations in neuronal vulnerability and demyelination in the internal granular, Purkinje, and molecular layers in cerebella of twitcher mice (Smith et al., 2014). Heterotopic Purkinje cells are typically observed in neurodevelopmental and neurodegenerative diseases, such as spinocerebellar ataxia type 1/6 or essential tremor, and are defined by a mislocalized cell body in the molecular layer (Kuo et al., 2011). In the normal cerebellum, Purkinje cells form a monolayer between molecular and granular layers, with their proper anatomical location as a requirement for physiological cerebellar functioning. In a disease characterized by severe cerebellar degeneration, such as KD, it is thus possible that aberrant Purkinje cell layer architecture contributes to ataxic symptoms.

### Combination approaches for treatment of KD

**Figure 9.**
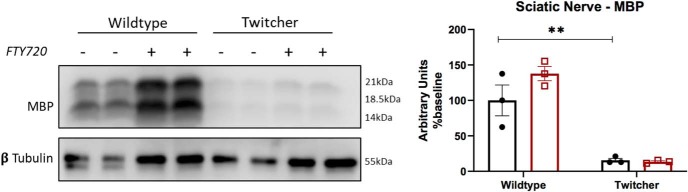
Myelin basic protein changes in the sciatic nerve of twitcher mice. MBP was used as a surrogate marker for measuring myelin levels in the sciatic nerves. As expected, there was a significant decrease in MBP levels in untreated twitcher mice (*p* = 0.0068, *n* = 3), which was not altered by fingolimod administration (*p* = 0.0058, *n* = 3). These findings thus suggest that fingolimod was not able to modulate peripheral nerve demyelination. Graphical data are represented as the mean ± SEM. **p < 0.01.

Our results show that fingolimod treatment increased the life spans of twitcher mice by up to 5 d and intermittently attenuated phenotypic abnormalities. While disappointing, these modest improvements may be explained by ongoing disease progression in the peripheral nervous system and other visceral organs. In supplementary studies, we observed little to no improvements in sciatic nerve demyelination in twitcher mice that were administered fingolimod (Fig. 9). Furthermore, we noted a consistent decrease in the size of visceral organs such as spleen and kidneys of twitcher mice, compared with healthy controls (data not shown). These observations imply that fingolimod-mediated improvements in the CNS are not sufficient to correct for the multiple organ failure, weight loss, and peripheral degeneration. Weight measurements of fingolimod-treated twitcher animals ultimately converges back to that of untreated littermates, and animals eventually develop tremors, flaccid paralysis of the hindlimbs that likely results from failed correction of peripheral nerve function, and demyelination. In support of this, failure to correct peripheral dysfunction in KD is considered a challenge in infants treated with presymptomatic hematopoietic stem cell transplantation (Escolar et al., 2005; Wenger et al., 2016). Thus, our observations, as well as those in previous studies, highlight the need to target both central and peripheral systems to provide a more effective therapy in KD (Lin et al., 2005; Rafi et al., 2012).
